# Perovskite Oxides: Syntheses and Perspectives on Their Application for Nitrate Reduction

**DOI:** 10.1021/acsomega.4c01487

**Published:** 2024-04-23

**Authors:** Ajibola A. Bayode, Odunayo T. Ore, Esther A. Nnamani, Babajide Sotunde, Daniel T. Koko, Emmanuel I. Unuabonah, Brigitte Helmreich, Martins O. Omorogie

**Affiliations:** † College of Chemical Engineering, Sichuan University of Science and Engineering, Zigong 643000, P. R. China; ‡ Department of Chemical Sciences, Faculty of Natural Sciences, Redeemer’s University, P.M.B. 230, 232101 Ede, Nigeria; § Department of Chemical Sciences, Achiever’s University, P.M.B. 1030, 341101 Owo, Nigeria; ∥ Environmental Science and Technology Unit, African Centre of Excellence for Water and Environmental Research (ACEWATER), Redeemer’s University, P.M.B. 230, 232101 Ede, Nigeria; ⊥ Chair of Urban Water Systems Engineering, School of Engineering and Design, Technical University of Munich (TUM), 85748 Garching, Germany

## Abstract

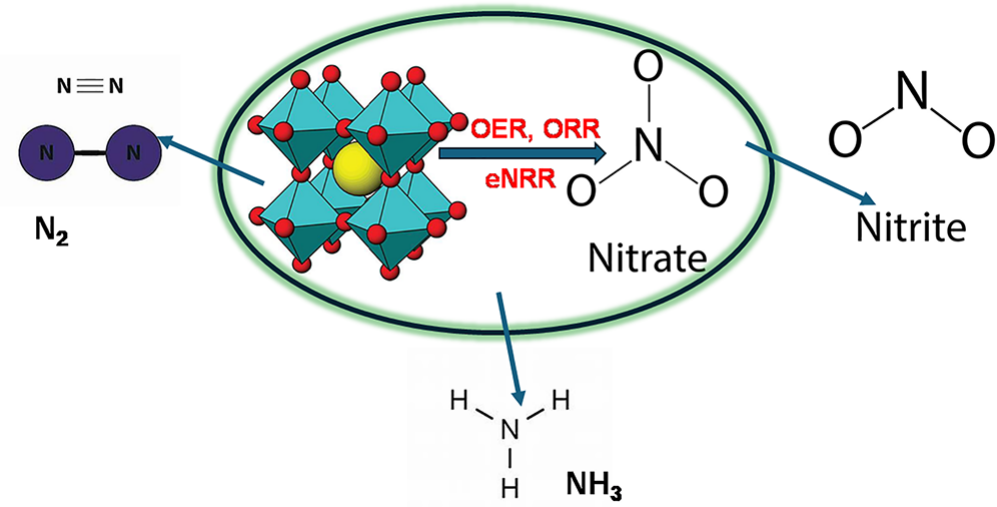

Over the decades, the rise in nitrate levels in the ecosystem has posed a serious threat to the continuous existence of humans, fauna, and flora. The deleterious effects of increasing levels of nitrates in the ecosystem have led to adverse health and environmental implications in the form of methemoglobinemia and eutrophication, respectively. Different pathways/routes for the syntheses of perovskites and their oxides were presented in this review. In recent times, electrocatalytic reduction has emerged as the most utilized technique for the conversion of nitrates into ammonia, an industrial feedstock. According to published papers, the efficiency of various perovskites and their oxides used for the electrocatalytic reduction of nitrate achieved a high Faradaic efficiency of 98%. Furthermore, studies published have shown that there is a need to improve the chemical stability of perovskites and their oxides during scale-up applications, as well as their scalability for industrial applications.

## 1 Introduction

Over the decades, nitrate compounds have been widely deployed to serve domestic, industrial, and agricultural purposes. Nitrate is an inorganic ion formed because of proton loss from nitric acid and is also the conjugate base of this acid. A molecule of nitrate consists of a nitrogen atom covalently bonded to three oxygen atoms and has the empirical formula NO_3_^–^. However, organic forms of nitrates have the general molecular formula RONO_2_, where R is the molecular formula of any organic compound.^1^ They are a class of ester compounds formed by the reaction of nitric acids with alcohols.^1^

Nitrates have been found useful in feeding ruminant animals and plants through nitrogen-based fertilizers. They have also served as essential human nutrients when ingested within safe consumption limits. For industrial purposes, nitrate compounds are used as strong oxidizing agents for manufacturing explosives, as fertilizers for enhancing plant growth, and as feedstock for processes where nitrate sources are required.^2^ Animals are fed with nitrate compounds as a major nitrogen source to make ammonia. Intake of human diets with nitrates helps raise nitric oxide levels in the human bloodstream, which helps regulate blood pressure, heart disease management, and regulation of the nervous systems as well as cardiovascular systems.^3^ Also, adequate nitrate intake has been identified to help improve eye condition and reduce the risk of age-related sight deficiencies when reduced into nitrite and nitric oxide, which are important nutrients to the eyes.

Their occurrence in human bodies mainly results from the consumption of vegetables and livestock animals. However, the ingestion of nitrates beyond the safe limit would lead to nitrate poisoning. Nitrate poisoning can be evident after plants are fed with nitrogen-based fertilizers.^4^ It is also evident in ruminant animals following how they consume nitrate-poisoned plants. However, nitrate poisoning could become evident in man because of poor air quality, water pollution, and even consumption of nitrate-poisoned plants and animals.

Nitrate deposits are found in both soil and water bodies.^5^ The accumulation of nitrates in soil results majorly from the use of excessive fertilizer, plant decomposition, deposition of animal waste, and runoffs from septic tanks.^2,6^ However, the high solubility of nitrates in water allows them to infiltrate into groundwater or enter surface water through erosion.^7−9^ Hence, wastes from domestic, agricultural, and industrial activities influence the level of nitrates found in ground and surface water.^2^ Effluents from poorly treated sewage plants would also be passages for excessive nitrate migration into our water bodies.^10^

Plants are the major sources of nitrates and are only found in minute concentrations in animal-based food products. However, nitrate concentration in food sources is generally higher than the observed concentration in water sources.^5,11^ Vegetable plants contain significant concentrations of nitrates, and nearly 80% of human diets are sourced from vegetable consumption.^5^

When nitrates accumulate in water bodies, they become a potential threat to both man and animals who primarily consume water,^12^ and health issues such as eutrophication, cancer and methemoglobinemia could result afterward.^4^

The abuse of nitrate intake poses a peril of cancerous diseases to both humans and livestock, as it becomes a cancer vector when ingested excessively. The toxicity of nitrates is measured by the quantity and rate at which it is being ingested. Nitrate ingestion from plant sources (mainly vegetables), animals (meats), and drinking water sources have varying toxic ingestion levels. The accumulation of nitrates in plants has been identified as the root of excessive nitrates ingestion by both man and livestock. This accumulation is a sequel to the effect of low rainfall and unfavorable sunlight conditions that impede the nitrate reductase system from performing significant conversion of nitrates to nitrites.^13^

The accumulation of nitrates in plants harms ruminant animals as it tends to raise the toxicity index of nitrites in their body system. The toxicity index of nitrite will tend to increase when the rate at which nitrates are converted into nitrites becomes much lower than the rate at which nitrites are converted into much-needed ammonia.^14^ The absorption of excess concentration of nitrites into the bloodstream of animals impedes the oxygen capacity of their red blood cell, leading to the formation of methemoglobin, a condition that could potentially lead to the termination of their life cycles.

A study reported that there may be a connection between excessive nitrates ingestion from plant vegetables and the development of cancerous growths in the pancreas of humans, but not with ingestion from drinking water sources.^15^ On the contrary, the cause of cancer issues related to the bladder was traced to nitrates ingestion from drinking water sources rather than vegetable plant sources.^16^ Another study reported that excessive ingestion of nitrates from processed meats may pose the risk of development of cancerous growth in the pancreas, bladders, and breasts of humans who consume them.^15,17,18^

The ingestion of excessive nitrates from drinking water sources is potentially harmful to both man and livestock. A study reported that women ingesting excessive quantities of nitrates from drinking water sources are prone to suffering cancerous growth in their ovaries.^19^ Another study has reported that excessive ingestion of nitrates from drinking water sources raises the tendency of the occurrence of gastric and colon cancers.^2,7^

Infants (≤6 months) are prone to suffer a health condition known as methemoglobinemia, because of ingestion of nitrates from drinking water sources beyond the safe limit.^2^ Pregnant women, as well as women trying to get pregnant, are equally prone to the risk of methemoglobinemia health condition as a result of excessive nitrates ingestion, allowing for malformation of babies in the womb and even undue termination of pregnancies.^20^

The World Health Organisation (WHO) and the United States Environmental Protection Agency (USEPA) guidelines have both established 0–50 mg·L^–1^ of nitrates concentration as safe limits for both humans and livestock.^11,20^ In contrast, a daily limit of 3.7 mg·kg^–1^ of nitrates intake has been recommended by the WHO, to avoid excessive nitrates intake.^5^

Nitrates being one of the main essentials of plants, animals, and human diets, cannot be done away with. However, conversion into other innocuous gases such as nitrogen and ammonia can manage their excessiveness. This can be achieved via direct decomposition, selective catalytic reduction, and nitrate storage and reduction methods.^21^

Nitrate reduction processes are essential to environmental sustainability because they reduce nitrate pollution which can have detrimental impacts on ecosystem and human health. One of the main byproducts of nitrate reduction is nitrite (NO_2_^–^). Sadly, nitrite is poisonous to aquatic life as well as people.^22^ However, as a fertilizer for plants, ammonium (NH_4_^+^) has several advantages. However, too much ammonium can cause eutrophication in water bodies, which can upset aquatic ecosystems and result in algal blooms.^23,24^ Nitrate reduction may also result in the release of nitrous oxide (N_2_O), a potent greenhouse gas that contributes significantly more to global warming than carbon dioxide.^25^ Emissions of nitrogen oxide contribute to both climate change and the depletion of the ozone layer. Nitrogen gas (N_2_), a harmless and inert gas, is produced when nitrate reduction is completed.^26,27^ Alternatively, an incomplete or inefficient reduction process may cause intermediary nitrogen species, such as nitrite, nitrous oxide (N_2_O), and nitric oxide (NO), to accumulate.^28^ These intermediaries may have detrimental effects on the ecosystem. Improving management strategies via the optimization of process parameters and reducing emissions can mitigate adverse environmental effects and promote the long-term adoption of nitrate reduction technology.^29^

Since nitrates are very soluble in water, the N=O covalent bond thus requires a considerably low energy to break.^12^ Some of the technologies that have been deployed to achieve nitrate reduction include photocatalytic, biological, and physical methods.^30^ Other treatment methods include electrodialysis, ion exchange, and biochemical treatment.^4^ However, the drawbacks associated with some of these techniques have resulted in researchers embracing electrochemical reduction as a more viable alternative for nitrate reduction.^4^ These drawbacks include the cost of pretreatment for electrodialysis, accumulation of brine wastes after ion exchange, and inconvenient operating conditions for biochemical processes.^4^

Electrochemical reduction has been reportedly appraised to be highly efficient and easy to deploy, with lesser demand for adding other chemicals when compared to the techniques mentioned above.^4^ It has also been reported that electrochemical reduction is a more viable green route for achieving biological denitrification and industrial production of ammonia.^10,12^ Also, the electrochemical reduction technique allows for selectivity in either producing nitrogen gas or ammonium, thus providing room for control and flexibility over the desired outcome.^31^ However, the technique is combated with the limitation of poor adsorption of nitrates and the capacity for activating adsorbed nitrates.^12^ The efficiency of the electrocatalytic process has been reported to be largely influenced by the rational design of electrocatalysts.^32,33^ Some electrocatalysts reportedly used for the electrochemical reduction of nitrates include noble metal electrodes, non-noble metal catalysts, bimetallic electrodes, metal compound electrodes, nonmetallic electrodes, and metal-molecular solid catalysts.^10^

However, compared to all of the stated electrocatalysts, perovskite oxides, with general molecular formula ABO_3_, have been reportedly identified and appraised for their capacities for achieving significant nitrate reduction, owing to their abilities to facilitate much adsorption of nitrates and significant reduction of the nitrates, which they do by providing more oxygen vacancy sites and allowing partial substitution of their B-site transition metals.^4,10^ It has been stated that perovskite oxides can be generally prepared by microemulsion, spray-drying, freeze-drying, citrate complexation, coprecipitation and sol–gel process.^34^ While many researchers have appraised perovskites for their capacity to achieve significant nitrates reduction in both lean and rich phase processes, some blocks have been identified as plagues that limit the efficacy of perovskites for electrochemical reduction of nitrates.

Thus, this review reports a comprehensive investigation of the facile syntheses and application of perovskite oxides for nitrates reduction and highlights a research focus geared toward improving the catalytic activity of perovskites for the reduction of nitrates.

## 2 Perovskites and Perovskite Oxides

The development of photocatalyst materials is one of the most promising and thriving answers for a clean and sustainable future, considering its cleanliness, inexhaustibility, efficiency, and affordability. Significant attempts have been made to create enormously operative photocatalyst materials for various applications, including removing carbon dioxide and nitrogen from the air and oxidizing organic water pollutants. In light of this, perovskite photocatalyst materials have gained special attention due to their exceptional properties because of their flexibility and adaptability in chemical composition, structure, bandgap, oxidation, and valence states.^35^

Perovskites are binary metal oxides with a general formula ABO_3_, where A cation can be a lanthanide, alkaline, or alkaline earth cation, and B cation is a metallic element with 3-, 4-, or 5-day configuration.^36^ There are several perovskite-related structures on the earth.^37,38^ This is due to their special structural physicochemical characteristics, such as hydrothermal stability, electron mobility, and REDOX behavior. As a result of these distinct features, perovskite and perovskite-related materials have emerged as an important new class of materials, making them very resourceful materials in catalysis, water splitting, solar cells, optical devices, and superconductors.^39^ Additionally, perovskite oxides can be used in various processes, including those that are liquid at ambient temperature, gas or solid at high temperatures, or under irradiation conditions.^40^

Perovskite oxides are compounds consisting of two or more simple oxides having high melting points.^41^ In their ideal form, perovskite oxides are cubic or nearly cubic, like other transition metal oxides containing the same formula (ABO_3_).^42^ Phase transitions may happen in some materials at low temperatures. Perovskite oxides have vast potential for many applications due to their structures and crystals, which are simple and exceptional in their ferroelectric and dielectric properties.^42,43^

**Figure 1:**
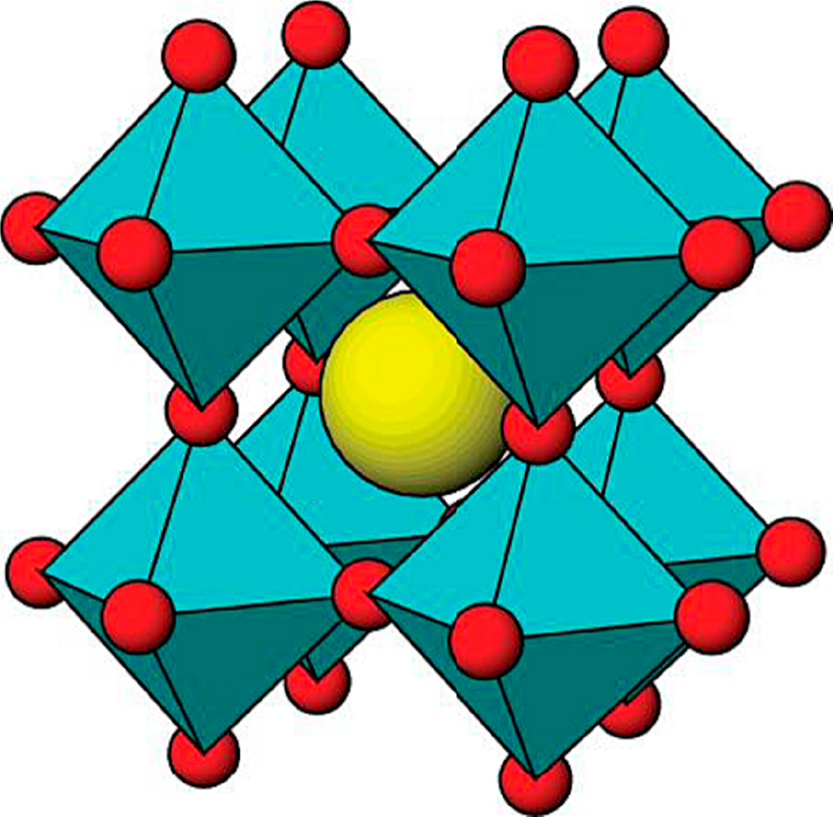
A diagrammatic representation showing the ideal cubic perovskite ABO3 structure (cyan, BO6 units; yellow, An atom; red, O atoms).^44^ Reprinted with permission from ref (44). Copyright 2012 American Chemical Society.

A 3-dimensional framework of BO_6_ octahedral that share their corners makes up the cubic cell (Figure 1). According to Kubacka et al.,^44^ Peña and Fierro,^45^ and Huang et al.,^46^ the B-site cation is a transition metal component. A group 2 or a rare earth element frequently makes up the A-site cation, which resides in the 12-coordinate location created by the BO_6_ network. As opposed to the model 3-dimensional perovskite ABO_3_, perovskite-related structures show lattice distortion to varying degrees and result in nonideal structures of the crystal phases like orthogonal, rhombohedral, tetragonal, monoclinic, and triclinic phases. These structures are caused by losing one or more proportional operators in the three-dimensional structure. Although the idealized structure is primitive cubic, the structure can be altered because of the radii of the two cations, which usually involves tilting of the BO_6_ units, also known as octahedral tilting.^44,47−49^

There are three forms of perovskite materials: the first has localized electrons, the second has delocalized energy-band states, and the third can switch between the first two. Perovskite structures come in a variety of forms, including A_2_BO_4_ layered Perovskite, ABO_3_ Perovskite, A_2_A_0_ B_2_B_0_′O_9_, Perovskite three and A_2_BB0′O_6_ Perovskite two.^50^ However, the most abundant ones are MgSiO_3_ and FeSiO_3._^43^ Oxides and oxides-like types of perovskite have different properties, such as insulator–metal transition, ionic conduction characteristics, dielectric, variation of solid-state phenomena, and metallic and superconducting characteristics.^43^

The structure could have various charge distributions depending on the cations’ potential valence and the material’s electroneutrality. Therefore, more than 90% of the metallic elements in the periodic table can be used to form perovskite structures.^51^ One of the advantages of perovskite structures is the possibility to adopt a wide range of different compositions, changing either the A or the B cation or being partially substituted by other cation(s) of the same or different valence, resulting in a general formula of A_1–*x*_A′_*x*_B_1–*y*_B′_*y*_O_3±δ_ that can adjust the REDOX, surface, and bulk properties.^52,53^ The stability of the structure depends directly on the geometrical constraints of octahedral or dodecahedral cavities.^54^ Also, the compounds with a formula of AB_2_O_4_ (A_3_O_4_ when A = B), which is recognized as a spinel structure, possess relatively similar physicochemical properties and are widely used together with ABO_3_ for catalysis applications due to their high activity and stability.^55^

The applications of perovskite oxides in heterogeneous catalysis can be dated back to the 1950s by Parravano, who reported the catalytic performance of NaNbO_3_, KNbO_3_, and LaFeO_3_ for CO oxidation.^39,56^ The first publications on perovskites, dated the 1970s, reported the exceptional catalytic properties in oxidation reactions and nitric oxide (NO) reduction, suggesting the possibility of replacing platinum-group metals with perovskite in automotive exhaust catalytic converters.^54^

### 2.1 Classification of Perovskites and Perovskites Oxides

Perovskites and perovskites oxides classification using anion X as the baseline. They include inorganic oxide perovskites, halide perovskites, hydride perovskites, and perovskites hydroxide.^42,43^

Another important classification is made based on the radii of their metallic ions.^43^ There are multiple perovskite-based combinations with different physical properties due to the ABO_3_ perovskite’s malleable crystal structure, capacity to accept a wide variety of cations in various oxidation states, and ability to accept cation or anion vacancies. These features lead to the formation of two key types of oxide phases, namely the ternary ABO_3_ type and their solid solutions and the complex modern type compounds (AB′*_x_*B″*_y_*) O_3_, where B′ and B″ are two separate elements in numerous oxidation states and *x* + *y* = 1. Subsequently, according to their oxidation states, ternary oxides can be further categorized into oxygen and cation-deficient species and A^1+^B^5+^O_3_, A^2+^B^4+^O_3_, A^3+^B^3+^O_3_.^57^

## 3 Synthesis of Perovskites and Perovskites Oxide Materials

Perovskites are usually formed at increased temperatures because, from their composition, perovskite oxides are compounds consisting of two or more simple oxides having high melting points.^58,59^ The technique used to synthesize perovskite oxide must be selected according to the specific application, specific demands of activity, and selectivity since these depend on how the atoms are arranged on its surface.^60^ It is worth stating that the synthesis techniques also affect the crystal structure and morphology of the synthesized samples.^61^ Consequently, the synthesis pathways can be categorized into three primary divisions: solid-state, liquid-state, and gas-state synthesis. Each method has a distinctive approach. Solid-state methods are used to synthesize bulk materials, liquid-state techniques are used to produce nanomaterials, and gas-state methods are mostly used to fabricate thin films.^62^

### 3.1 Solid-State Synthesis Technique

The solid-state synthesis method is commonly used to prepare perovskite in pure form due to the availability of impurity-free precursors, and they find key applications in electronic industries. Researchers most frequently employ this method.^58^ Also, most ceramics are evenly synthesized in this way and are utilized to create polycrystalline materials. This method requires raw carbonate and/or oxide form materials.^63^ The raw components do not interact chemically in this process at room temperature. However, the chemical reaction occurs quickly when the raw material mixture is heated to very high temperatures (about 700–1500 °C). The downside of the solid-state synthesis approach is that it requires annealing at high temperatures for a long time and frequent intermediary grindings which results in poor homogeneity and difficulty controlling the particle size.^58,62^ Therefore, the problem arises when perovskites from solid-state methods are subjected to surface-related studies.^64^ The different synthesis routes using the solid-state technique are highlighted below.

#### 3.1.1 Mechanical Ball-Milling Method

The mechanical ball-milling method produces solid-state perovskite compounds in bulk. The raw materials are oxides and/or carbonates, which are hand-mixed, ball-milled, and calcined at a high temperature to form perovskite.^59,62^

#### 3.1.2 High-Speed Ball-Milling Method

The high-speed ball-milling and the mechanical ball-milling methods are very similar. However, the striking distinction between the two techniques is that high-speed ball milling uses a very high rotation per min (rpm). The technique also uses low temperatures to create nanoparticles.^65^ Due to the high likelihood of chemical reactions occurring during high-energy ball-milling, which could produce a variety of harmful gases, this approach only uses metal oxides.^62,66^

### 3.2 Liquid-State Synthesis Technique

The liquid-state synthesis is designed to make nanomaterials. Researchers and scientists most frequently employ it to create nanoparticles of oxide materials.^67^ Typically, this method utilizes raw materials in nitrates, acetates, or oxalates form. They are combined and expected to react with one another at room temperature. Autocombustion, sol–gel, and coprecipitation are some of the different liquid-state synthesis techniques used to prepare perovskite nanomaterials, which are to be described in more detail.^43^

#### 3.2.1 Autocombustion Method

**Figure 2:**
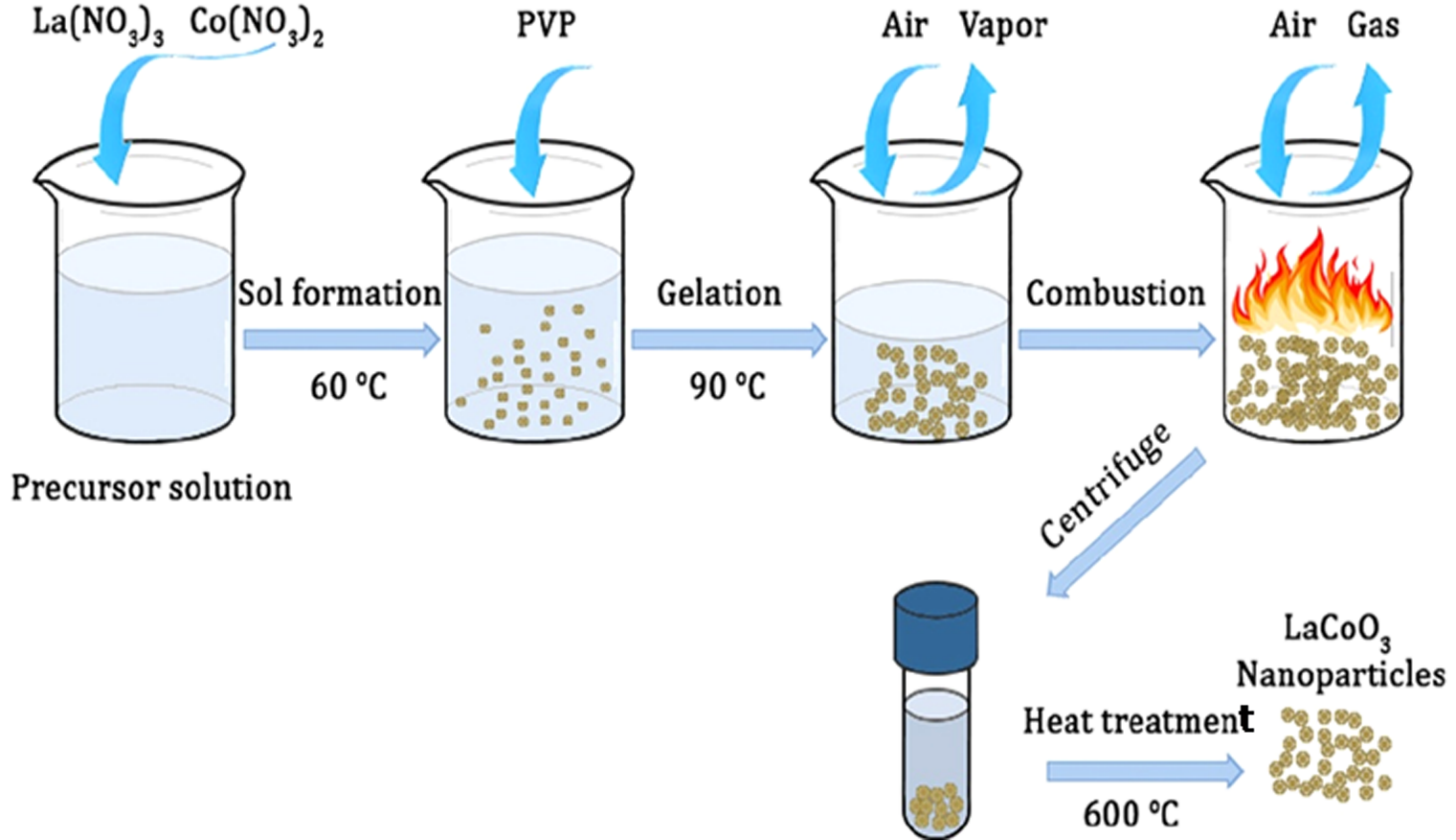
Schematic diagram of the synthesis of LaCoO_3_ perovskite oxide using the Autocombustion method.^70^ Reprinted with permission from ref (70). Copyright 2021 John Wiley & Sons Ltd.

The autocombustion method is an easy, low-cost approach for producing perovskite nanomaterials. The starting materials for this method are oxalates, acetates, or nitrates, which are readily soluble in deionized water.^62^ It uses some organic fuel to aid in combustion, such as urea, citric acid, and glycine. In a work by Kumar et al.,^68^ the starting materials La_2_O_3_, SrCO_3_, and Mn(CH_3_COO)_2_·4H_2_O were utilized to create La_0.7_Sr_0.3_MnO_3_ perovskite Manganite, with glycine serving as the fuel. La_2_O_3_ and SrCO_3_ were first prepared as nitrates by dissolving them in dilute nitric acid, followed by the dissolution of Mn (CH_3_COO)_2_·4H_2_O and glycine in distilled water to start the reaction. Each of the materials’ precursor solutions was independently dissolved and synthesized. At the end, they were mixed while continuously stirred in a big beaker, that was heated on a magnetic stirrer at 175–200 °C.^68^ After 6–7 h of continuous spinning, the mixed solution thickened and changed into a gel-like structure as seen in Figure 2. As the stirring time increased, an autocombustion occurred, resulting in a flame that emitted a vast array of distinct gases. The temperature of the entire combination increased to 800–1000 °C within a short period during the igniting process. The produced blackish-brown powder was removed from the beaker and separated into several portions for calcination at varied temperatures.^69^

#### 3.2.2 Sol–Gel Method

**Figure 3:**
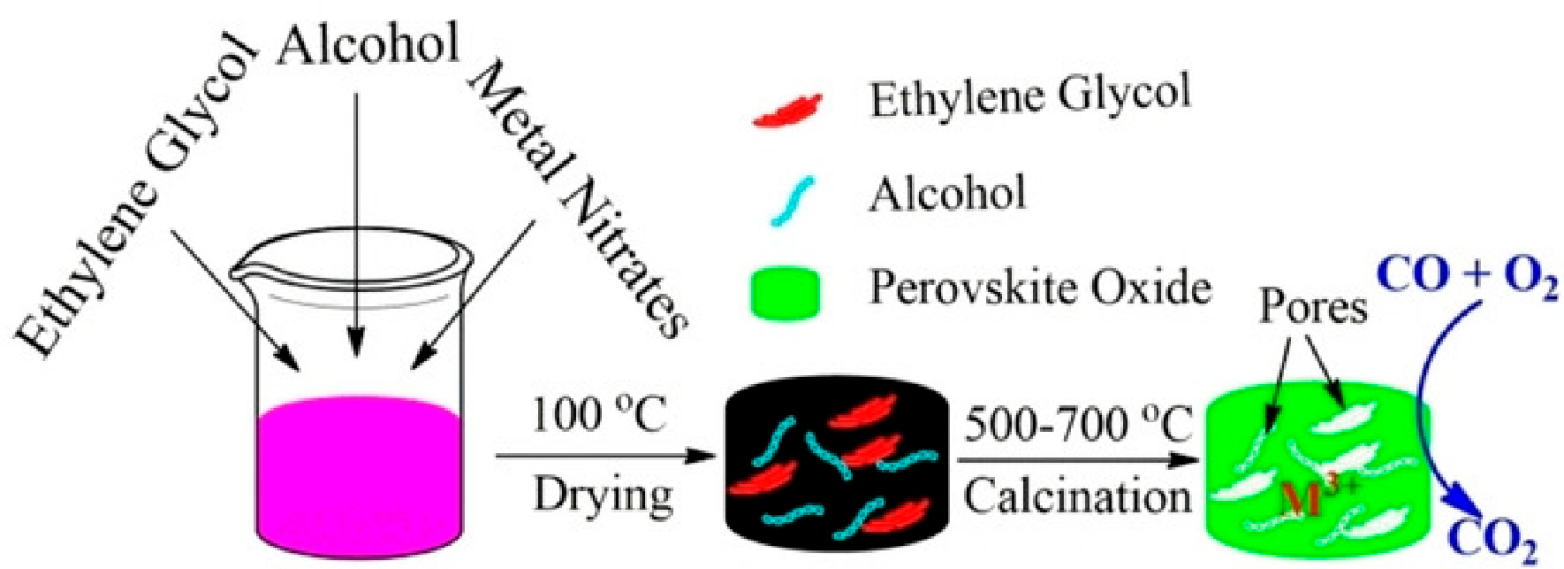
Schematic representation of sol–gel synthesis method for LaCoO_3_ perovskite oxide.^75^ Reprinted with permission from ref (75). Copyright 2018 John Wiley & Sons Ltd.

Most chemists produce nanomaterials using the sol–gel technique. This method includes both physical and chemical processes related to the following: hydrolysis, condensation, polymerization, gelation, drying, and densification.^71^ This technique uses metal alkoxides as starting materials.^72^ Metal alkoxides typically have a chemical formula of M(OR)_*x*_. They are assumed to be either a derivative of alcohol ROH, where R is an alkyl group or a derivative of metal hydroxide M(OH)_*x*_.^62,73^ A mole ration of metal alkoxides is measured and melted in alcohol or deionized water at 60–80 °C under continuous swirling. It is crucial to regulate the pH value of metal alkoxide solutions to prevent precipitation and to create a homogeneous gel produced by basic or acidic solutions.^72^ Hydrolysis and condensation are two terms used to describe the entire process, which results in the production of polymeric chains. A gel eventually forms because of the development of the polymeric chains, which also causes a noticeable increase in the reaction mixture’s thickness as shown in Figure 3. To avoid undesired substances, the gel must be dried between 150 and 200 °C. The resultant gel was annealed at various temperatures between 400 and 800 °C after removing the contents to produce the pure phased materials.^64,72^ For instance, Andrade et al.^74^ synthesized nanotubes and nanoparticles of La_0.6_Ca_0.4_MnO_3_ perovskite Manganite using the sol–gel method following calcination at different temperatures.^74^ The mole ratio of La(NO_3_)_3_·6H_2_O, CaCO_3_, and Mn(CH_3_COO)_2_·4H_2_O were utilized for the production of La_0.6_Ca_0.4_MnO_3_ perovskite. To initiate the process, CaCO_3_ was dissolved in nitric acid and converted into CaNO_3_, while La (NO_3_)_3_·6H_2_O and Mn(CH_3_COO)_2_·4H_2_O were dissolved in distilled water. The combinations of all precursors were done using a beaker. As a polymerizing agent polyethylene glycol (PEG) was added in the proper quantity to the precursor solutions. The solution was heated at 70 °C for 6 h to complete the polymeric development. The solution eventually changed into a thick, yellow gel that could be calcined from 700 to 1000 °C.

#### 3.2.3 Coprecipitation Method

The coprecipitation method requires metal cations from a general medium and precipitates as oxalates, carbonates, citrates, or hydroxides as raw materials.^62,64^ To get the unadulterated phase in the polycrystalline form, the resulting precipitates will have to be calcined at various temperatures after being washed many times with distilled water. With this technique, nearly homogeneous polycrystalline powders can be obtained. For proper precipitation, the solubility of the compounds utilized should be relatively close to one another.^76^ It is essential to remember that the precursor solutions should be mixed at the atomic level to create smaller particles and be calcined at low temperatures to produce a pure material.^77^ Also, the controlling pH of the precursor solution, stirring speed, concentration, and mixture temperature are vital parameters for the coprecipitation method.^78^

**Figure 4:**
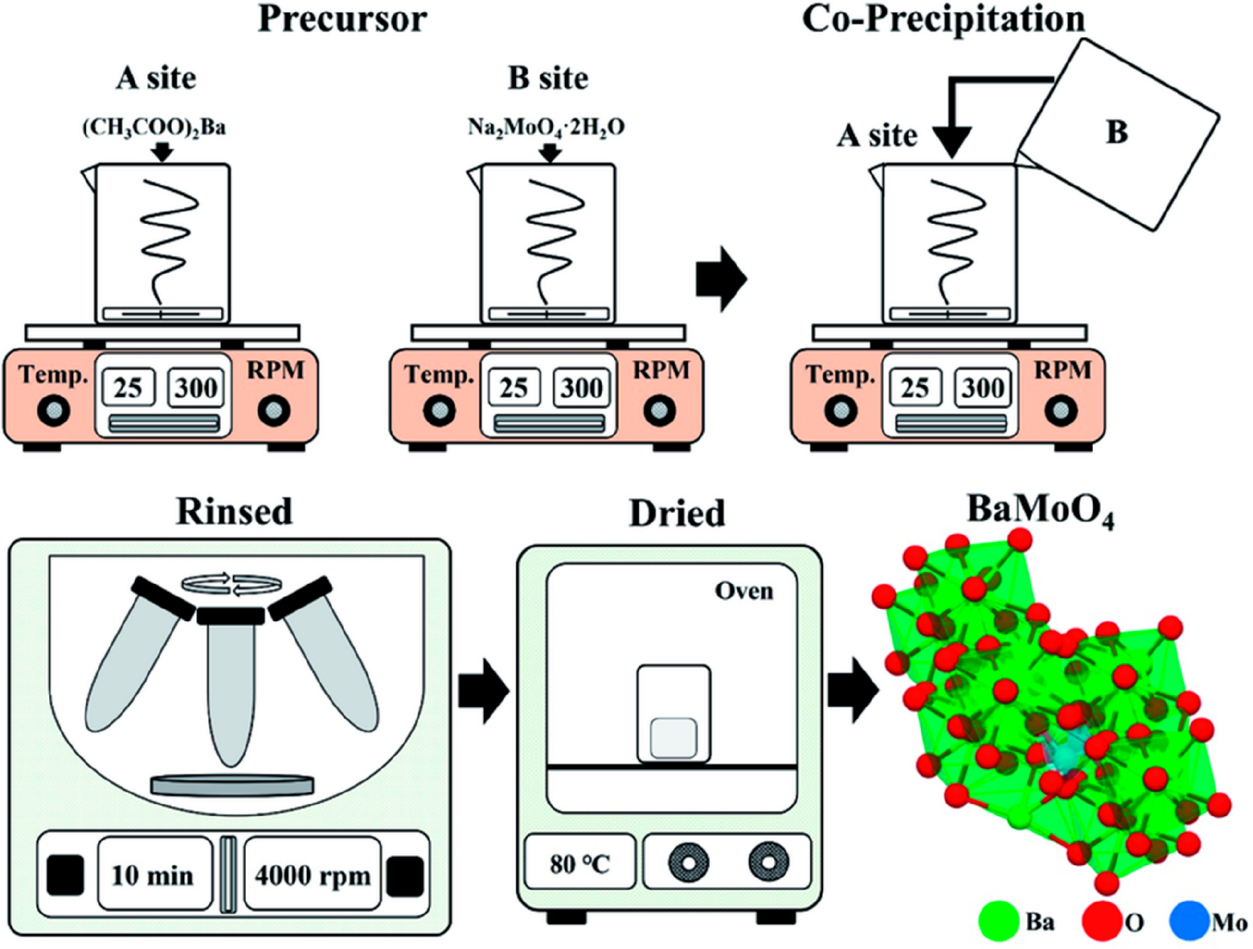
Schematic representation of the synthesis of BaMoO_4_ through the coprecipitation method.^81^ Reprinted with permission from ref (81). Copyright 2022 Royal Society of Chemistry.

The LaMn_1–*x*_Fe_*x*_O_3_ (*x* = 0, 0.1, 0.2) perovskite synthesized by Geetha et al.^79^ is an example of the coprecipitation method.^79^ Mole ratio of La(NO_3_)_3_·6H_2_O, Fe(NO_3_)_3_·9H_2_O and MnCl_2_·4H_2_O were dissolved in distilled water. These solutions were combined in a single platform and stirred continuously at 50 °C for 30 min. Immediately, NaOH solution was introduced slowly into the mixture until the pH of the solution got to 13.0. The combined solution of the precursors was stirred continuously until the creation of a black precipitate as shown in Figure 4. The precipitate was collected and treated with distilled water several times to remove excess chlorides, and oven-dried at 50 °C. Consequently, the finished product was calcined for 6 h at 800 °C. Usually, the liquid-state technique at very low temperatures is used to form nanoparticles from perovskite materials. However, submicron-sized perovskite materials can be created by burning them at a higher temperature, similar to the solid-state technique.^80^

### 3.3 Gas-State Synthesis Technique

The gas-state synthesis technique is a bottom-up method for synthesizing multifunctional nanoparticles.^82^ The gas-state synthesis method uses a variety of processes, including lasers, furnaces, flames, and plasmas, to synthesize powdered oxide materials. Although their reactors are different, the fundamentals of thermodynamics and kinetics of the reaction are highly similar.^62^ The method of bottom-up nanofabrication is based on gathering nanomaterials from smaller components.^62^ These methods provide a fine dispersal of the nanoparticles. The dispersion must be reduced for the narrow distribution of the nanoparticles as it increases in the particle size.^83^ Coevaporation of Y, Cu, and BaF_2_ was used to create YBa_2_Cu_3_O_7_ films, which were subsequently annealed at high temperatures in a moist O_2_ environment with water vapor to speed up the annealing process and minimize substrate contact.^64^

There are various techniques for the preparation of thin films, such as chemical vapor deposition, molecular beam epitaxy, laser ablation, direct current (DC) sputtering, magnetron sputtering, thermal evaporation, and electron beam evaporation.^62,84,85^ These methods are entirely distinct from other synthesis methods and are used to generate thin films for various electronic gadgets and solar cells made of perovskite materials.^86^ For these synthesis techniques to provide the appropriate properties in the generated perovskite materials, an exact setup for high-quality samples is required.^87^ Gas-state synthesis techniques are divided into three groups, namely; synthesis at the temperature of crystallization in an appropriate environment, fabrication at a temperature between 500 and 800 °C, followed by a postannealing process at a higher temperature, and fabrication with the substrate heated to a very high temperature afterward for postannealing.^43,64,87^

The perovskite materials can be produced utilizing gas-state processes for a range of purposes, including photocatalysts, solar cells, optical and anticorrosion coatings, capacitor dielectrics, semiconductor devices, bioimplantable devices, chemical reactors, and catalysts.^43,62^ In the approaching years, industrial interest will undoubtedly rise in creating nanomaterial-based technologies through gas-state synthesis.^88^

### 3.4 Electrospinning Method

Electrospinning is a widely used technique for the synthesis of perovskite oxide nanofibers. This approach offers a promising way to tailor the properties and applications of perovskite oxides. These oxides, which have an ABX_3_ crystal structure, have gained significant attention due to their diverse functionalities, including catalysis, sensing, energy storage, and electronic devices.^89^

To employ electrospinning for perovskite oxide synthesis, a precursor solution containing the metal cations (A and B-site elements) and the oxygen source required for perovskite oxide synthesis is prepared. The compatibility of the electrospinning process with the precursor salt and solvent should be considered while preparing the solution. The precursor solution is then loaded into a syringe equipped with a fine needle or spinneret, which is connected to the high-voltage power supply. A high voltage is applied to the precursor solution using the power supply, inducing electrostatic forces that draw the solution from the syringe tip toward the grounded collector. As the solution travels through the air, solvent evaporation occurs, leading to the formation of perovskite oxide nanofibers.^90,91^

Following the electrospinning process, the as-spun perovskite oxide nanofibers are typically subjected to thermal treatment or annealing at elevated temperatures. This process is crucial for the crystallization of the perovskite structure and the removal of residual solvents, resulting in well-defined oxide nanofibers. By using electrospinning for perovskite oxide synthesis, researchers can achieve precise control over the morphology, structure, and properties of oxide nanofibers. This approach enables the development of advanced materials with tailored functionalities and applications. Moreover, the scalability and versatility of the electrospinning technique make it a promising approach for large-scale production of perovskite oxide nanofibers for various industrial and technological applications.^92,93^Table 1 shows the merits and demerits of various routes of perovskite oxide, with respect to the conditions required to optimize them.

**Table 1:** Advantages and Disadvantages of Different Synthesis Routes of Perovskite Oxide

synthesis route	advantages	disadvantages
sol–gel method	•It is reproducible.	•Some precursor materials used in the sol–gel method may exhibit poor stability or reactivity, leading to difficulties in controlling the synthesis process and obtaining the desired perovskite oxide structure.
	•The reaction is simple and easy.	
	•The sol–gel process allows precise control over the composition of the perovskite oxide by adjusting the precursor concentrations and ratios. This enables the synthesis of complex compositions and doping with various elements to tailor the properties of the materials.	•While the sol–gel method is suitable for laboratory-scale synthesis, scaling up production for industrial applications may be challenging due to issues such as reproducibility, uniformity, and cost-effectiveness.
	•The sol–gel method typically results in homogeneous mixtures at the molecular level, leading to a uniform distribution of components within the final perovskite oxide material. This uniformity enhances the material’s properties and performance.	•The sol–gel process often requires prolonged heating or annealing steps, leading to higher energy consumption compared to some other synthesis methods. This can increase production costs and environmental impact.
	•By adjusting parameters such as solvent type, pH, and drying conditions, the sol–gel method enables control over the morphology and microstructure of the perovskite oxide, which is crucial for optimizing its performance in different applications.	
autocombustion method	•It is simple and cost-effective.	•Autocombustion synthesis may not be easily scalable for large-scale production due to the inherent limitations of the combustion process and the need for careful control over reaction conditions.
	•The combustion process can result in highly homogeneous materials with fine particle sizes and uniform distribution, leading to improved properties and performance of perovskite oxides.	•The combustion process involves complex chemical reactions, which can lead to challenges in controlling the reaction kinetics and product formation, potentially resulting in nonuniform or undesirable material properties.
	•This method can yield high-purity materials because the combustion process can effectively remove impurities through the formation of gaseous products.	•Autocombustion synthesis involves combustion reactions, which can be hazardous if proper safety precautions are not followed, particularly due to the release of flammable gases and potential for uncontrolled reactions.
	•Autocombustion synthesis offers good control over the stoichiometry of the synthesized perovskite oxide, leading to precise tuning of material properties.	•This method may not be suitable for synthesizing certain types of perovskite oxides or achieving specific material structures or morphologies, depending on the precursor chemistry and reaction conditions.
		•The as-synthesized materials may require additional processing steps, such as calcination or annealing, to improve crystallinity, phase purity, or other material properties, adding complexity to the overall synthesis process.
electrospinning method	•Electrospinning enables precise control over the morphology of perovskite oxide materials, allowing for the fabrication of nanofibers, nanotubes, or other nanostructures with high aspect ratios and specific surface areas.	•It produces nanofibers with limited thickness, which may restrict the application of perovskite oxide nanofibers in certain contexts where thicker structures are required.
	•The electrospinning process can produce highly uniform and continuous nanofibers, leading to enhanced material properties and performance compared to bulk counterparts.	•Achieving optimal electrospinning conditions for synthesizing perovskite oxide nanofibers can be challenging and often requires careful optimization of parameters such as solution viscosity, flow rate, applied voltage, and collector configuration.
	•Electrospinning can be combined with various precursor materials, dopants, and processing parameters to tailor the composition, structure, and properties of perovskite oxide nanofibers for specific applications.	•Perovskite oxide nanofibers synthesized via electrospinning may require additional post-treatment steps, such as calcination or sintering, to enhance crystallinity, phase purity, and material properties, adding complexity to the synthesis process.
	•The nanofibrous structure produced by electrospinning results in a high surface area-to-volume ratio, which is beneficial for applications such as catalysis, sensing, and energy storage.	•Electrospinning typically results in randomly oriented nanofibers, which may limit control over the alignment and orientation of perovskite oxide nanofibers, particularly for applications requiring specific structural arrangements.
	•Electrospinning is a scalable and relatively straightforward technique, making it suitable for large-scale production of perovskite oxide nanofibers compared to other synthesis methods.	•Electrospinning setups can involve complex equipment and instrumentation, including high-voltage power supplies, syringe pumps, and specialized collectors, which may require expertise and investment in infrastructure for implementation and operation.
ball milling	•Ball milling can achieve high efficiency in the synthesis of perovskite oxides by promoting rapid mixing and reaction between the precursor materials due to the intense mechanical forces generated during milling.	•Ball milling requires high energy input due to the intense mechanical forces involved in the process, which can lead to increased energy consumption and equipment wear, particularly for prolonged milling durations or hard precursor materials.
	•Ball milling ensures uniform mixing of precursor powders, leading to homogeneous distribution of components and improved stoichiometry control in the synthesized perovskite oxide materials.	•Ball milling can introduce contaminants from the milling media (e.g., balls and vials) or atmosphere (e.g., oxygen, moisture) into the synthesized perovskite oxide materials, affecting their purity, phase composition, and properties.
	•Ball milling can reduce the particle size of perovskite oxide precursors to the nanoscale, resulting in increased surface area and enhanced reactivity, which is beneficial for achieving desired material properties.	•Ball milling may result in nonequilibrium phases or crystal defects in the synthesized perovskite oxide materials, limiting control over crystal structure and potentially affecting material performance.
	•Ball milling can be applied to various types of perovskite oxide precursors, including oxides, carbonates, and nitrates, allowing for the synthesis of a wide range of perovskite oxide compositions and structures.	•Ball milling can generate heat during the milling process, which may lead to temperature rise in the milling vial and the synthesized perovskite oxide materials, affecting reaction kinetics, phase formation, and material properties.
	•Ball milling is a scalable process suitable for both laboratory-scale research and large-scale production of perovskite oxide materials, making it a versatile and widely applicable synthesis technique.	•Ball milling can cause particle agglomeration due to the adhesive forces between particles, leading to the nonuniform distribution of components and reduced effectiveness in achieving desired material properties.
coprecipitation method	•Coprecipitation is a relatively simple and cost-effective method for synthesizing perovskite oxides compared to other techniques, requiring minimal equipment, and operating under mild conditions.	•Coprecipitation reactions can be relatively slow, requiring long reaction times to complete the precipitation process and achieve the desired phase formation and crystallinity in the synthesized perovskite oxide.
	•Coprecipitation typically yields perovskite oxide powders with high purity because the precipitation process effectively removes impurities through the formation of insoluble precipitates.	•Coprecipitation may result in particle agglomeration or the formation of large aggregates due to the presence of high surface energy and the tendency of particles to coalesce during drying and calcination.
	•Coprecipitation allows for precise control over the stoichiometry of the synthesized perovskite oxide by adjusting the ratio of precursor salts in the solution, leading to tunable material properties.	•Coprecipitation often produces perovskite oxide powders with irregular shapes and sizes, limiting control over the morphology and surface properties of the synthesized materials compared to other synthesis methods.
	•Coprecipitation can produce highly homogeneous perovskite oxide powders with fine particle sizes and uniform distribution of components, resulting in improved material properties and performance.	•The as-precipitated perovskite oxide powders may require additional processing steps, such as calcination or annealing, to improve crystallinity, phase purity, and material properties, adding complexity to the synthesis process.
	•Coprecipitation is easily scalable for large-scale production of perovskite oxide powders, making it suitable for industrial applications	•Coprecipitation reactions are sensitive to reaction conditions such as temperature, pH, and stirring rate, which may require careful optimization to achieve reproducible results and desired material properties

## 4 Electrocatalytic Reduction of Nitrates by Perovskites

Conventional physicochemical technologies comprising biochemical treatment, ion exchange, and electrodialysis have been employed for nitrate reduction.^94^ However, these technologies are confronted with certain drawbacks, including harsh operating environments, brine wastes after ion exchange, and the pretreatment requirements for electrodialysis. These limitations have hindered the large-scale applications of these technologies.^95^ In contrast, the minimal input of chemicals, moderate operating conditions, and high efficiency of electrochemical reduction have made it a promising method over the aforementioned technologies. The electrocatalytic reduction of nitrate is a cost-effective and environmentally friendly method because the nitrate undergoes reduction by protonated hydrogen or electrons without using other reducing agents.^94^ Studies have shown that electrocatalysis has excellent potential for removing nitrate. It offers the relative advantages of easy operation and simplicity of reactor structure.^96,97^

The efficiency of electrocatalytic reduction can be improved by adjusting the parameters as well as the appropriate selection of suitable electrode materials. A suitable electrode material should be characterized by good corrosion resistance, high stability, high catalytic activity, and low cost.^98,99^ In many studies, metal cathode materials such as Pd, Pt, Cu, Co, Fe, Ni, and Ti have been developed to efficiently reduce nitrate.^100−109^ However, the use of these metal cathode materials is impaired by drawbacks owing to high toxicity and high cost.^99^

Due to their low cost, flexible structure, and remarkable catalytic activity, perovskite materials have been thoroughly studied as alternatives to noble metal-based electrocatalysts.^110^ They are essential to the effort to achieve a sustainable energy future. Numerous important reactions, including the reduction of oxygen, the evolution of oxygen, and the evolution of hydrogen, are catalyzed by these materials in electrocatalysis.^111^ For instance, to ensure the sustainable synthesis of ammonia via the electrochemical reduction of nitrate, a study employed the use of bismuth ferrite (BiFeO_3_) as an electrocatalyst.^112^ The study demonstrated that deformed perovskite-type bismuth ferrite (BiFeO_3_) flakes are excellent catalysts for the electrochemical production of NH_3_ via nitrate reduction. At a voltage of −0.6 V versus the reversible hydrogen electrode, they achieved an NH_3_ yield of 90.5 mg·h^–1^·mg_cat_^–1^ and a maximum Faradaic efficiency of 96.9%.^112^ The design and optimization of perovskite-based catalysts through ongoing research and development will propel substantial advancements in sustainable nitrate reduction technology. These developments could lead to more effective and environmentally friendly cleanup techniques.

Metal-modified biochar with advantageous physical and chemical surface characteristics has demonstrated great potential in the adsorption of water contaminants, including phosphate and nitrate. This is principally accomplished by interactions with the oxygen-containing functional groups on the surfaces of the biochar and/or ion exchange mechanisms. Metal-modified biochar may be promising in the electrocatalytic reduction of nitrate.^113^ This led Liu and his colleagues to adopt a typical perovskite/biochar nanocomposite, LaFeO_3_/biochar, as a photocatalyst in a study owing to its strong magnetism, narrow band gap, and high stability.^114^ By copyrolyzing Lotus biomass and Fe/La salts, composites of LaFeO_3_ and biochar-rich in defective oxygen and surface functional groups were successfully produced. There was no usage of organic reagents during this process, which could have been hazardous to the environment. The subsequent photocatalytic reduction of nitrate to ammonia was carried out using the resultant nanocomposites. The study results in changed structural and surface characteristics of the catalysts through interactions with lanthanum (La^3+^) and iron (Fe^3+^) ions. The oxygen defects in LaFeO_3_ were enhanced by adding biomass, hastening the electron–hole pair separation process. Simultaneously, Fe/La salts contributed to the surface alteration of the biochar during the carbonization process, increasing the exposure of aromatic structures and functional groups that contain oxygen, which promoted nitrate adsorption. Crucially, the REDOX-active quinone/phenol groups on the surface of the biochar promoted the selectivity of ammonium ion (NH_4_^+^) as a direct electron donor by helping with the exchange of photogenerated electrons. Using the LaFeO_3_/biochar photocatalyst under visible light irradiation, nitrate conversion reached 98%, and ammonia selectivity reached 97% when the mass ratio of lotus and Fe/La salts was optimized.^114^ An innovative method for reducing nitrate is to combine metal-modified biochar with perovskite-based nanocomposites. By utilizing the unique qualities of both materials, this synergistic combination improves catalytic performance and environmental sustainability. Such composite materials have a great deal of potential for cost-effective and scalable nitrate remediation, particularly in decentralized or resource-constrained environments where traditional treatment methods would not be practical.

Also, a highly effective catalyst is essential to achieving high conversion and selectivity in the electrochemical reduction of nitrate. The high activity of these catalysts allows them to accelerate the electrochemical reduction reaction, increasing the rate at which nitrate is converted into the desired product.^115^ Additionally, they exhibit excellent selectivity, which minimizes the production of undesirable byproducts while directing the reaction toward the intended product.^115^ These effective catalysts reduce overpotentials, suggesting that the reaction needs less energy. This improves the process’s energy efficiency, which is important for industrial applications. The sustainability of the process is equally enhanced while reducing its environmental impact.^116,117^ This probably inspired the work of some researchers who assembled a perovskite (LaFeO_3_) on hydrothermal carbonation carbon (HTCC) to obtain a nanostructured photocatalyst that was used for the reduction of nitrate to ammonia.^118^ The two-dimensional HTCC nanosheet has several surface functional groups and a substantial specific surface area. Fe/La salt was used to modify the surface of HTCC, increasing the aromatic structure and the exposure of oxygen-containing functional groups and promoting nitrate adsorption. Furthermore, a p–n heterojunction between HTCC and LaFeO_3_ was created, facilitating the quick separation of photogenerated electron holes and improving photocatalytic activity. Under the effect of visible light irradiation, the LaFeO_3_/HTCC photocatalyst obtained a peak nitrate removal of 94.6% and an ammonia selectivity of 88.7% when the mass ratios of pomegranate peel to Fe/La salt were optimized.^118^

While the catalytic activities and tunable physicochemical properties of perovskites have made them resourceful in the reduction of nitrates, their design strategies have been predominantly focused on the selection of B-site cations to enhance the reduction process through the mechanisms of Mars-van-Krevelen-like, Langmuir–Hinshelwood-like, or Eley–Rideal pathways which usually proceed on metal sites.^119−121^ However, recent observations have shown that modification strategies can be employed to improve the efficiency of electrocatalytic nitrate reduction. One such strategy involves doping metal cation on the B-site of perovskite, which was adopted in a study by Zhang and his Colleagues.^122^ The study employed the doping of Mn cation on the B-site of LaCoO_3_ and investigated its performance in the electrochemical reduction of nitrate. LaMn_0.6_Co_0.4_O_3_, the optimized doped material, showed a nitrate removal efficiency of 41.9%, greater than LaCoO_3_. The study reported that the doped material exhibited outstanding stability after 10 consecutive reaction cycles and performed well under various operating conditions, such as pH levels, cathode potentials, and varying initial nitrate concentrations. It was deduced that the doped Mn cation not only affected the valence of the Co cation but also activated adsorbed oxygen to give an electron and speed up electron transfer based on the observed changes in the valence of Co and Mn in the cathode before and after electrocatalysis.^122^

Furthermore, it has been suggested that the activity of perovskite is widely influenced by the electronic environment around the active sites.^123^ Perovskite oxides exhibit a remarkable degree of compositional flexibility since they can accommodate about 90% of the metallic elements found in the periodic table. As a result, metal elements can be integrated to produce a bimetallic perovskite at the B-site. The catalytic performance of these perovskite oxides can be altered based on band theory and molecular orbital theory. This can be accomplished by modifying their [BO_6_] units’ octahedral structure, managing the hybridization of B–O bonds, and generating oxygen vacancies. For example, it has been established that a critical component of electrocatalytic activity is the covalency of the bonds between transition metals and oxygen, which reflects the adsorption strength of intermediates connected to oxygen.^124,125^ In this regard, Chu and his Colleagues attempted to optimize the adsorption strength of intermediates by constructing a series of Fe-rich perovskite oxides of LaFe_0.9_M_0.1_O_3-δ_ (where M = Cu, Ni, and Co) from the starting material LaFeO_3−δ_ (LF) by a B-site substitution strategy.^126^ The results of the study showed that the LaFe_0.9_Cu_0.1_O_3−δ_ (LF_0.9_Cu_0.1_) submicrofibers showed a superior ammonia yield rate of 349 ± 15 μg·h^–1^·mg_cat_^–1^, and a Faradaic efficiency of 48 ± 2%, compared to LF submicron fibers. These submicrofibers have a more robust Fe–O hybridization, an increased number of oxygen vacancies, and a more positive surface potential.^126^

Moreover, oxygen vacancies have been reported to enhance the selectivity and efficiency of the electrochemical reduction of nitrate. The N–O bond in nitrate can be efficiently activated by oxygen vacancies, which improves the yield and selectivity of the nitrate reduction process to ammonia.^127^ Also, by modifying the local electronegativity and coordination environment, oxygen vacancies added to the structure of catalysts for oxygenated compounds can enhance the catalytic property of the nitrogen reduction process.^128^ Hence, an attempt to improve the performance of electrochemical nitrate reduction could incorporate the engineering of oxygen vacancies. It traps metastable electrons in the antibonding orbitals of nitrogen molecules, breaking the N≡N bond and facilitating fast electron transport.^129^ In a study by Feng et al.,^12^ NbWO_6_ perovskite nanosheets with oxygen vacancy were investigated in the selective electro-reduction of nitrate. In this study, thermal treatment and exfoliation were used to create NbWO_6_ nanosheets with an oxygen vacancy (NbWO_6–*x*_), which showed an NH_3_ selectivity of 86.8% and a Faradaic efficiency of 85.7% toward the electrocatalytic reduction of nitrate. Using ^1^H nuclear magnetic resonance spectra and ^15^N isotope labeling tests, the origin of NH_3_ from NO_3_ was verified. Computational studies were conducted to reveal the role of the oxygen vacancy in the electrocatalytic reduction of nitrate.^12^ Similarly, another study by Yang and his colleagues aimed to improve the performance of perovskite oxides by oxygen vacancies engineering.^130^ The study presented a novel and effective electrochemical activation technique for the in situ synthesis of oxygen vacancies (OVs). The results showed that the activated La_0.9_FeO_3−δ_ had a NO_3_^–^–N removal rate that was 2.6 times higher than the unmodified La_0.9_FeO_3-δ_. The greater adsorption energy of NO_3_^–^ and the facilitation of the synthesis of atomic hydrogen (H*) for the hydrogenation of NO_3_^–^–N were credited to the increased presence of OVs, which also improved the performance of the nitrate reduction reaction. Furthermore, a-240 h continuous experiment showed that the activated La_0.9_FeO_3-δ_ remained exceptionally stable.^130^ In addition, the electrochemical reduction of nitrate can be improved by A-site deficiency engineering of the electrocatalysts. This was reflected in a study by Liu and colleagues.^110^ The study presented a practical method for modifying the A-site deficiencies in cobalt-based perovskite oxides to increase nitrate electro-reduction activity (NO_3_ER). To demonstrate the concept, the authors used a sequence of (Ba_0.5_Sr_0.5_)_1–*x*_Co_0.8_Fe_0.2_O_3−δ_, where *x* = 0, 0.05, 0.10, 0.15, and 0.20. Their NO_3_ER activity peaked at *x* = 0.15 and showed a volcano-type reliance on the *x* values. To be more precise, (BS)_0.85_CF outperformed most previously reported NO_3_ER catalysts in terms of activity (143 mg·h^–1^·mg_cat_^–1^ or 0.86 mmol·h^–1^·cm^–2^), selectivity (97.9%), and stability (200 h) at −0.45 V (vs. RHE). The optimized properties of NO_3_ER can be explained by the modulation of physicochemical properties caused by A-site inadequacies, including the introduction of a modest distance between the Fermi level and the band center and the production of a moderate amount of oxygen vacancies.^110^

The potential for improving the catalytic efficiency of perovskite oxides in nitrate reduction is highlighted by the application of modification techniques such as metal doping and oxygen vacancy engineering. A more thorough comprehension of the fundamental mechanisms driving these modifications will make catalyst design more accurate and efficient.

Recently, the construction of perovskite oxides with sufficient active sites for the electrochemical reduction of nitrate has seemed to be challenging. This has brought about the adoption of high entropy materials. High entropy materials are particularly useful in electrocatalysis owing to random element distribution, complex electronic structures, and intrinsic abundant active sites.^131^ While various high entropy materials including phosphate, carbides, and nitrides have been developed, high entropy perovskites seem to be generating considerable interest due to their structural stability, several active sites, and tunable constituent elements.^132^ Chu and his colleagues investigated high-entropy perovskite oxides as electrocatalytic nitrogen reduction reaction (eNRR) catalysts. These oxides have the composition Ba_*x*_(FeCoNiZrY)_0.2_O_3−δ_, where *x* = 0.9 and 1. The materials produced more oxygen vacancies at the A-site by changing the non stoichiometric metal components. Particularly, high-entropy perovskite oxides exhibited a markedly increased eNRR activity. Specifically, compared to B(FCNZY)_0.2_, the NH_3_ yield and Faraday efficiency for B_0.9_(FCNZY)_0.2_ were 1.95 and 1.51 times greater, respectively.^131^ This underscores the importance of designing and developing novel high entropy perovskite oxides with advanced microstructures that will simultaneously accelerate the reactions as well as increase the number of active sites on the perovskite oxides.

Because of their high conductivity and stability, transition metal oxides have found extensive use as electrocatalysts.^133^ However, their Faradaic efficiency is frequently constrained, particularly in oxygen evolution reactions (OER). Unwanted byproducts may be produced during OER as a result of side reactions.^134^ In contrast, perovskite oxides show better Faradaic efficiency in OER, as evident in the studies earlier reported. Their high surface area and adjustable electronic structure reduce side reactions and improve selectivity for the intended oxygen evolution process, which is why they are relatively superior to other materials. Perovskite oxides are attractive options for effective electrocatalysis because of these characteristics. In addition, the catalytic activity of noble metals, such as platinum (Pt) and gold (Au), in a variety of electrochemical reactions is widely recognized. Unfortunately, their high price and limited availability prevent them from being widely used.^135,136^ Furthermore, even though noble metals have a high Faradaic efficiency, they can deactivate and become poisonous in harsh operating conditions.^137,138^ Perovskite oxides offer an appealing alternative. In certain processes, such as oxygen reduction reactions (ORR), they provide similar or even better Faradaic efficiency. In addition, perovskite oxides are more resilient and prevalent than noble metals. Their favorable cost-performance ratio makes them an attractive option for long-term electrocatalysis. Also, the high surface area and tunable features of carbon-based materials, such as graphene and carbon nanotubes, have attracted a lot of interest as electrocatalysts.^139^ However, heterogeneous architectures and surface defects might reduce their Faradaic efficiencies, leading to unpredictable catalytic behavior.^140^ Perovskite oxides, however, have surface chemistry that can be controlled and well-defined crystalline structures. They consequently show improved stability and increased Faradaic efficiency in a range of electrochemical processes. Perovskite oxides are potential options for dependable and effective electrocatalysis because of their characteristics. Finally, metal sulfides and selenides have become more and more popular as electrocatalysts owing to their abundance and advantageous catalytic characteristics.^141,142^ However, slow kinetics and unfavorable side effects have the potential to hinder their Faradaic efficiency.^143,144^ Perovskite oxides provide a higher Faradaic efficiency due to their unique electronic structure and composition. They do this by promoting charge transfer and reducing energy losses.^145−147^ Consequently, in a number of electrochemical processes, perovskite oxides perform better than metal sulfides and selenides.

The preceding studies highlighted several ways to modify perovskite materials to increase their electrocatalytic performance for nitrate reduction. These include the creation of oxygen vacancies, metal doping, and engineering of A-site deficiencies. Various approaches demonstrated how flexible perovskite structures are and how they may be tailored for specific catalytic applications. Most of the studies emphasized how perovskite-based electrocatalysts for nitrate reduction provide great catalytic efficiency and selectivity. Many studies highlighted the environmentally favorable features of synthesis methods, like using biomass to create perovskite-based catalysts and avoiding dangerous organic reagents. These factors highlighted the potential of perovskite electrocatalysts to support environmentally friendly activities and are consistent with the growing emphasis on green and sustainable chemistry. Nevertheless, despite the strong magnetism, narrow band gap, and great stability displayed by perovskites like LaFeO_3_/biochar, further investigation is still required to determine their long-term stability under a range of operating conditions. The mass ratio of biomass to metal salts is one of the many variables that can impact the performance of perovskite/biochar composites. Meticulous adjustments are required for these parameters to function at their best. Even though research on a laboratory scale has produced promising results, it is still unclear whether these approaches can be scaled up to industrial levels.

## 5 Future Perspectives and Conclusions

In this review, we provided an overview of nitrates and their toxicity. Additionally, we discussed the use of perovskites and perovskite oxides for nitrate reduction. We looked at the electrocatalytic nitrate reduction mechanism, including surface modifications, metal doping, and the importance of oxygen vacancies. However, to develop better catalysts, we need a deeper understanding of their fundamental mechanisms, such as the exact pathways of the reduction events and how perovskite structures interact with nitrate ions. Perovskite oxides have shown high catalytic activity in nitrate reduction reactions, indicating their potential to remove nitrates from water sources effectively. Selectivity toward desired products is crucial in nitrate reduction reactions to avoid the formation of harmful intermediates. Perovskite oxides usually have good selectivity toward nitrogen gas, which is a harmless end product. Stability of the catalysts over extended periods is essential for practical applications. Perovskite oxides have shown good stability under certain conditions, but further research is necessary to optimize stability in different environments.

Although the reviewed studies’ findings are promising, it is crucial to identify potential obstacles and areas that require further research. We must consider the catalyst’s stability across multiple reaction cycles, its scalability for industrial use, and the toxicity of specific metal ions. Additionally, more research is necessary to determine the durability and long-term performance of perovskite-based electrocatalysts in real-world applications. In the future, researchers could combine perovskite-based electrocatalysts with other technologies, such as advanced sensing and monitoring systems, to increase the overall efficiency of nitrate reduction processes. This would provide real-time feedback and control, maximizing the efficiency of reaction conditions while minimizing the generation of undesirable byproducts.

To implement perovskite oxides for nitrate reduction in real-world situations, we must evaluate their economic feasibility and scalability. Factors such as the availability of raw materials, synthesis costs, and scalability of production methods must be considered. We must also assess the overall environmental impact of using perovskite oxides for nitrate reduction, including energy requirements for synthesis, potential byproducts, and the process’s overall sustainability. Integrating perovskite oxide-based catalysts into current water treatment systems or developing new systems is necessary to ensure practical applicability. In conclusion, although perovskite oxides show promise as catalysts for nitrate reduction, further research is necessary to address stability, scalability, cost-effectiveness, and environmental impact challenges before widespread implementation. Collaborative efforts between researchers, industry, and policymakers are essential to realize the full potential of perovskite oxide-based catalysis for nitrate reduction and effectively mitigate nitrate pollution.
